# Genome-wide identification and characterization of *LcCCR13* reveals its potential role in lignin biosynthesis in *Liriodendron chinense*

**DOI:** 10.3389/fpls.2022.1110639

**Published:** 2023-01-16

**Authors:** Wei Li, Ziyuan Hao, Lichun Yang, Hui Xia, Zhonghua Tu, Zhengkun Cui, Junpeng Wu, Huogen Li

**Affiliations:** Key Laboratory of Forest Genetics & Biotechnology of Ministry of Education, Co-Innovation Center for Sustainable Forestry in Southern China, Nanjing Forestry University, Nanjing, China

**Keywords:** Liriodendron chinense, lignin biosynthesis, cinnamoyl-CoA reductase, CCR gene family, expression analysis

## Abstract

**Introduction:**

Wood formation is closely related to lignin biosynthesis. Cinnamoyl-CoA reductase (CCR) catalyzes the conversion of cinnamoyl-CoA to cinnamaldehydes, which is the initiation of the lignin biosynthesis pathway and a crucial point in the manipulation of associated traits. Liriodendron chinense is an economically significant timber tree. Nevertheless, the underlying mechanism of wood formation in it remains unknown; even the number of LcCCR family members in this species is unclear.

**Materials and Results:**

This study aimed to perform a genome-wide identification of genes(s) involved in lignin biosynthesis in L. chinense via RT-qPCR assays and functional verification. Altogether, 13 LcCCR genes were identified that were divided into four major groups based on structural and phylogenetic features. The gene structures and motif compositions were strongly conserved between members of the same groups. Subsequently, the expression patterns analysis based on RNA-seq data indicated that LcCCR5/7/10/12/13 had high expression in the developing xylem at the stem (DXS). Furthermore, the RT-qPCR assays showed that LcCCR13 had the highest expression in the stem as compared to other tissues. Moreover, the overexpression of the LcCCR13 in transgenic tobacco plants caused an improvement in the CCR activity and lignin content, indicating that it plays a key role in lignin biosynthesis in the stems.

**Discussion:**

Our research lays a foundation for deeper investigation of the lignin synthesis and uncovers the genetic basis of wood formation in *L. chinense*.

## Introduction

Lignin is an aromatic phenolic compound formed by the polymerization of three monolignols (*p*-coumaryl alcohol, coniferyl alcohol, and sinapyl alcohol, also called the H, G, and S subunits) in plants. It is extremely abundant in nature and is only second to cellulose in the constituents of natural biomass (Li et al., 2009). Lignin plays a pivotal role in maintaining the cell structure and imparting resistance to biotic and abiotic stresses (Bhuiyan et al., 2009; Weng et al., 2010; Srivastava et al., 2015). Lignin content is also a critical factor in determining the specific applications of different types of wood. Wood with high lignin content is more rigid and usually used in furniture manufacture, while wood with low lignin content is easier to degrade and is often used in the pulp and paper industries (Boerjan et al., 2003).

Due to its agricultural and economic importance, the lignin biosynthesis pathway has been comprehensively investigated in the past. The phenylpropanoid pathway, an important pathway in lignin synthesis, requires the participation of a series of enzymes. Enzymes in this process are associated with 4-coumarate: CoA ligase (4CL), cinnamate-4-hydroxylase (C4H), phenylalanine ammonia-lyase (PAL), caffeic acid *O*-methyltransferase (COMT), hydroxycinnamoyl-CoA shikimate/quinate hydroxy-cinnamoyl transferase (HCT), cinnamate-3-hydroxylase (C3H), caffeoyl shikimate esterase (CSE), caffeoyl-CoA 3-*O*-methyl-transferase (CCoAOMT), cinnamoyl-CoA reductase (CCR), cinnamyl alcohol dehydrogenase (CAD), and ferulate-5-hydroxy-lase (F5H). Briefly, phenylalanine is converted to *p*-coumaroyl-CoA *via* PAL, C4H, and 4CL, where *p*-coumaroyl-CoA is the branching point in the flavonoid pathway (Vogt, 2010). F5H and COMT convert coniferaldehyde to sinapaldehyde, and CCR triggers the reduction of feruloyl-CoA to coniferaldehyde (Li et al., 2005; Leple et al., 2007; Vanholme et al., 2008; Yan et al., 2019). And CAD is involved in the last enzymatic step of monolignol biosynthesis by reducing the substrates, coniferaldehyde, and sinapaldehyde, to the G and S monolignols (Yan et al., 2019). Moreover, it has been reported that most CAD enzyme-coding genes can influence plant growth by participating in lignin biosynthesis. In *Gossypium hirsutum*, *Gh4CL*-silencing and -overexpressing plants have a ~ 20% reduction and a ~ 10% increase in lignin content (Sun et al., 2020). In *Populus trichocarpa*, monolignol biosynthesis is influenced by the PtrCAD1-PtrCCR2 protein complex (Yan et al., 2019).

The first step of the lignin reduction reaction requires cinnamyl-CoA reductase, which employs five hydroxyl cinnamic acid coenzyme CoA esters (*p*-coumaryl-CoA, caffeoyl-CoA, feruryl-CoA, 5-hydroxyeruloyl-CoA, and sinapyl-CoA) as substrates to catalyze the production of cinnamaldehyde, leading to the generation of lignin (Lewis and Yamamoto, 1990; Goujon et al., 2003). Therefore, the cinnamyl-CoA reductase encoded by the *CCR* gene is the point of initiation for the lignin synthesis pathway (Lacombe et al., 1997; Humphreys and Chapple, 2002; Larsen, 2004). The carbon flux toward lignin is regulated by the *CCR*-coding enzyme, thus, it is a suitable target to alter lignin levels (Lacombe et al., 1997). Due to their key roles in monolignol biosynthesis, *CCR*s have been cloned and characterized in many plants, including the monocotyledons, *Zea mays* (Pichon et al., 1998), *Triticum aestivum* (Ma, 2007), *Lolium perenne* (Tu et al., 2010), and *Lilium Oriental Hybrids* (Li et al., 2009), as well as the dicotyledons, *Caragana korshinskii* (Li et al., 2014), *Salvia miltiorrhiza* (Wang et al., 2012), *Arabidopsis thaliana* (Lauvergeat et al., 2001), *Populus tomentosa* (Chao et al., 2017), and *Betula platyphylla* (Wei, 2012), etc.

*CCR* genes are preferentially expressed in the stems or roots of plants (Pichon et al., 1998; Lauvergeat et al., 2001; Larsen, 2004), and have thus been thought to be involved in lignification. Moreover, manipulation of the *CCR* expression typically results in a large variation in the lignin content and composition. Plants with heavily down-regulated *CCR* expression often exhibit stunted growth and delayed development, along with altered carbon fluxes across lignin and other metabolic pathways (Yin et al., 2021). The overexpression of *BnCCR1* and *BnCCR2* has been reported to increase lignin content in stems and roots of *Brassica napus*, which improved the lodging resistance in transgenic *BnCCRox* lines (Yin et al., 2021). Su et al. reported that *PbCCR1/2* are related to lignin biosynthesis in overexpression transgenic plants (Su et al., 2019). A similar phenotype was observed in birch; Zhang et al. reported that the overexpression of *BpCCR1* increases lignin content in transgenic plants (Zhang et al., 2015). Giordano et al. demonstrated that the spatiotemporal expression pattern of *CCR1* cDNAs from *Paspalum dilatatum* correlates with the developmental profile of lignin deposition (Giordano et al., 2014). Compared to the wildtype (WT) plants, the lignin content in *A. thaliana* mutant *irx4* plants is established to reduce significantly (50% of that in the WT plants), leading to abnormal growth of the *irx4* plants (Smith et al., 2017). Based on previous studies, regulating the expression of the *CCR* gene may be an effective way to change the lignin content in plants. As more plant genome resources become available, genome-wide surveys will enable systematic characterizations of key enzymes and their corresponding family members. Identification and functional analysis of lignin biosynthesis enzymes and their associated genes will lay a foundation for the systematic analysis of carbon flux through lignin metabolism (Wang et al., 2019).

*Liriodendron*, a tertiary relic genus, belongs to the Magnoliaceae family. At present, there are only two natural species in this genus, *L. tulipifera* L and *L. chinense* (Hemsl.) Sarg. *L. tulipifera* is distributed throughout eastern North America, while *L. chinense* is scattered in southern China and northern Vietnam (Yang et al., 2021). Due to their fast growth rate, strong stress resistance, and good wood quality, *Liriodendron* trees are widely cultivated for use in timber, furniture, and paper-making industries. Lignin is closely related to wood quality, and *CCR* is a key enzyme-encoding gene in lignin synthesis (Chao et al., 2017). Nevertheless, little is known about the key genes involved in lignin biosynthesis in *L. chinense.* Therefore, this study aims to identify LcCCR gene family members in *L. chinense* and analyze their potential roles in lignin synthesis. Our results revealed that the *LcCCR13* gene participates in lignin synthesis and is useful for the elucidation of the mechanism of wood formation in *L. chinense.*

## Materials and methods

### Plant materials

All plant materials were obtained from a provenance test plantation of *L. chinense* in Xiashu Forest Farm, Jurong City, Jiangsu Province (119°13′E, 32°7′N), China. The provenance of the sample tree was the Lushan Natural Reserve, Jiangxi Province (116°0′E, 29°32′N). From March to July, 2021, we collected leaves, shoots, roots, petals, and stems, which were quickly frozen in liquid nitrogen and stored in the refrigerator at –80°C before RNA extraction.

*Nicotiana alata* seedlings were sterilized with 10% NaClO for 15 min and then sown in 1/2 MS medium. After 2 days of vernalization (4°C dark), the seedlings were placed in an incubator (22°C with 16-h light and 8-h dark photoperiod) for a week. They were then transplanted into a medium for culturing.

### Identification and characterization of *LcCCR*s

We downloaded the *Liriodendron* Genome resources from the NCBI database (https://www.ncbi.nlm.nih.gov/; PRJNA418360; Chen et al., 2019). Thirteen *AtCCR*s that were identified to be involved in the biosynthesis of lignin precursors in *A. thaliana* were obtained from the TAIR databases (https://www.arabidopsis.org/). Thirteen AtCCR proteins (**Table S1**) were used as alignment sequences to perform BLAT alignment with the protein database of *L. chinense*. The E-value was set to 0.001 to obtain the candidate *CCR* sequences of *L. chinense*. All candidate CCR sequences were assessed based on the presence of the conserved domain with InterPro (http://www.ebi.ac.uk/interpro/search/sequence/) and CDD search (https://www.ncbi.nlm.nih.gov/Structure/bwrpsb/bwrpsb.cgi) procedures. Finally, sequences with complete CCR domains were selected, and the sequences with more than 97% similarity between the different databases were deleted. The physical and chemical characteristics of LcCCR proteins identified from the *L. chinense* genome were predicted by ProtParam online tool (https://web.expasy.org/protparam/; Wilkins et al., 1999). The WoLF PSORT (https://wolfpsort.hgc.jp/) and TargetP-2.0 Server (http://www.cbs.dtu.dk/services/TargetP/) were used to predict the subcellular localization of the LcCCR proteins.

### Multiple alignment and phylogenetic analysis

To group the CCR proteins in *L. chinense*, we analyzed the phylogenetic relationships between *L. chinense* and other plants. We constructed a phylogenetic tree using the CCR protein sequences from *A. thaliana*, *P. tomentosa*, *L. perenne*, *Oryza sativa*, *Z. mays*, and *L. chinense* (**Table S1**). DNAMAN (version 6.0) software was used to perform multiple comparisons among these protein sequences, and a phylogenetic tree was constructed by the maximum likelihood method *via* MEGA (version 5.1) software. Substitution and site change rates were calculated using the Jones–Taylor–Thornton (JTT) model and the Gamma distributed with Invariant sites (G+I) model. Bootstrap analysis was performed with 1000 replicates to calculate the reliability of the phylogenetic tree. Finally, the network profile of the phylogenetic tree was visualized by Evoview (https://www.evolgenius.info/evolview/) (He et al., 2016). Last, we used DNAMAN (version 6.0) to compare the 13 candidate *LcCCR* genes with other bona fide *CCR* genes.

### Conserved motif and gene structure analysis

To analyze the exon-intron structure of *CCR* genes, the annotation profile was retrieved from the *L. chinense* genome (Chen et al., 2019). Information on the introns and exons of the LcCCR gene family was visualized by GSDS (version 2.0) (http://gsds.cbi.pku.edu.cn/) online tool (Hu et al., 2014). The conserved motifs in *LcCCRs* were visualized by MEME (version 5.3, https://meme-suite.org/meme/tools/meme) online tool (Bailey et al., 2006). The parameters were set as follows: an optimum motif could contain no less than 6 and no greater than 200 residues; the maximum number of motifs allowed was 10 (Chen et al., 2020b). The results of these motifs were then visualized *via* the TBtools software (Chen et al., 2020a).

### Prediction of *cis*-acting elements in the promoters of *LcCCRs*

To identify the *cis*-acting elements in the promoter sequences of the 13 *CCRs* in *L. chinense*, the 2000-bp upstream sequence of the start codon (ATG) in *CCRs* was analyzed. The types and numbers of *cis*-elements in *LcCCRs* were evaluated *via* the Plant CARE (https://bioinformatics.psb.ugent.be/webtools/plantcare/html/) online tool (Lescot et al., 2002).

### Expression profile analysis of *CCR* genes in different tissues

To quantify the expression levels of *LcCCR* genes in the different tissues (leaf, shoot apex, and developing xylem at the stem (DXS)), we analyzed the expression profiles based on the transcriptome data (Unpublished data from our laboratory). The RPKM (reads per kilobase per million mapped reads) approach was used to represent the expression abundance of each *LcCCR*s. The TBtools (version 1.0) software was used to draw the gene expression heatmap (Chen et al., 2020a).

### RNA extraction and RT-qPCR analysis of *CCR* genes

To identify genes involved in lignin biosynthesis, we conducted the RT-qPCR analysis of *CCR* genes in five tissues (leaves, shoots, roots, petals, and stems). The detailed protocols are as follows:

Total RNA was isolated from each sample using a total RNA isolation kit (TIANGEN, China) following the manufacturer’s protocol. RNA-free deoxyribonuclease (DNase I) was used to remove trace DNA from the extracted RNA. The integrity of the total RNA was detected *via* 1.0% agarose gel electrophoresis. The concentration and purity of the total RNA were analyzed by a NanoDrop 2000c spectrophotometer (Thermo Scientific, Wilmington, DE, USA). Reverse transcription was performed using the Evo M-MLV RT Premix for qPCR (AG11706, ACCURATE BIOTECHNOLOGY, HUNAN, Co., Ltd). Quantitative primers for the *CCR* genes were designed using Oligo 7.0 software according to strict requirements (**Table S2**). The RT-qPCR was run in a StepOnePlusTM System (Applied Biosystems) as a 10-μL reaction mixture containing 5 μL of 2× SYBR Premix Ex Taq, 0.2 μL of 50× ROX Reference Dye (SYBR Green Premix Pro Taq HS qPCR Kit (AG11701, ACCURATE BIOTECHNOLOGY, HUNAN, Co., Ltd)), primers, and cDNA. The *LcActin*97 (Tu et al., 2019) and *NtActin* (AJ421411) primers were used for the amplification of the internal reference gene. To ensure the accuracy of the results, three biological replicates (where each biological replicate had three technical replicates) were conducted, and the data were examined using the 2^−ΔΔCT^ method. Significance was determined by the *t*-test using the SPSS statistical software (version 20, IBM, New York, NY, USA; *p < 0.05, **p < 0.01).

### Function validation of *LcCCR13* using genetic transformation

*LcCCR13* was retrieved from the existing *L. chinense* genome data (Chen et al., 2019). We designed the intermediate fragment-specific PCR primers *via* the Oligo (version 7.0) software (**Table S2**). Reverse-transcribed cDNA was used as a template for PCR to synthesize intermediate fragments. The PCR products were cloned into the pEASY-Blunt Zero Cloning Kit (Transgen Biotech, Beijing, China) and transformed into *E. coli* (DH5α). Finally, the amplicons were sequenced by Jie Li Biology (Shanghai, China).

The sequenced ORF of the *LcCCR13* gene was inserted into the overexpression vector, which was digested with *Xba* I and *Bam*H I QuickCut enzymes (Takara Biomedical Technology, Dalian, China). The construct was then transformed into *Agrobacterium tumefaciens* strain EHA105 by the freeze-thaw method (Holsters et al., 1978, Zhang et al., 2012). The target gene was transferred into WT tobacco by the leaf-disc conversion method. Six transgenic tobacco lines featuring independent recombination events were identified by molecular detection; they were all heterozygous with similar phenotypes. To detect the relative expression levels of *LcCCR13* in the different lines, three transgenic lines together with the WT strain were subjected to a semi-quantitative PCR assay. The relative expression level of *LcCCR13* was quantified from three biological replicates, where each replicate had three technical replicates.

Meanwhile, the histochemical staining of stem segments from transgenic tobacco was performed *via* the Phloroglucinol-HCL and safranin staining methods (Yang et al., 2022). The stained sections were observed under a Zeiss Axio microscope. The ImageJ software (version 2.3.0) was then used to calculate the size of the thickened and lignified cells (μm).

All the transgenic and WT plants were acclimated and grown in a greenhouse at 18–23°C with 60% humidity under a 16-h light and 8-h dark photoperiod at NJFU (Nanjing Forestry University, Nanjing, China).

### Determination of plant height, CCR activity, and lignin content

The height and the diameter were measured in 2-month-old transgenic and WT plants. The measurements for each plant line included at least 5 replicates.

The CCR activity was determined following the method of Hamedan et al. (Hamedan et al., 2019).

According to the method of Bhaskara et al. (1999), a 10 mg powdered sample was weighed and ground into a homogenate in a mortar with 95% ethanol. The homogenate was then transferred to a 50-mL centrifuge tube for centrifugal separation at 4000 rpm for 5 min. The pellet thus obtained was washed twice with 5 mL of 95% ethanol and 6 mL of ethanol: n-hexane (1:2 (v/v)). The pellet was dried naturally. Six milliliters of 25% acetyl bromide solution in glacial acetic acid were used to dissolve the precipitate, followed by being covered and sealed in a water bath at 70°C for 30 min. Subsequently, 0.9 mL of 2 mol·L^−1^ NaOH, 5 mL of glacial acetic acid, 0.1 mL of 7.5 mol·L^−1^ hydroxylamine hydrochloride, and 15 mL of glacial acetic acid were added to the centrifuge tube. Instead of the substrate, distilled water was used for the same reaction as a control. The absorbance of the supernatant was measured at 280 nm using the GeneQuant pro ultraviolet spectrophotometer (Biochrom Ltd, Cambridge, UK), and the absorbance per gram of the dry sample at 280 nm represented the lignin content per gram (OD g^−1^ DW) (OD: Optical Density, DW: dry weight). The statistical significance of the data was estimated by analysis of variance (ANOVA); the Pearson coefficient was calculated using the SPSS statistical software (version 20, IBM, New York, NY, USA; *p < 0.05, **p < 0.01).

### Data processing and statistical analysis

The statistical significance of the data using ANOVA was assessed using the SPSS statistical software (version 20, IBM, New York, NY, USA). Multiple comparative analyses were performed using the *t*-test (*p < 0.05, **p < 0.01). To identify differences among the samples or plant lines. One-way ANOVA followed by Duncan’s multiple comparisons test was used.

## Results

### Genome-wide identification of the *LcCCR*s in *L. chinense*

Thirteen *LcCCR*s were identified in this study containing the complete “X-W-Y-X-X” functional domain. Detailed characteristics of the 13 *LcCCR*s are presented in **Table 1**. These *CCR* genes were named *LcCCR1-LcCCR13*. Briefly, the length of the coding DNA sequences (CDS) ranged from 786 to 1188 bp with 5–7 exons in each, and the amino acid length ranged from 261 (*LcCCR4*) to 368 (*LcCCR48*) amino acids, with the number of amino acids being greater than 300, except for in two proteins (*LcCCR4/6*). In addition, the molecular weight (MW) of the proteins ranged from 28933.98 (*LcCCR4*) to 43564.06 (*LcCCR11*) Da, where most protein’s MW was greater than 30.00 kDa. The isoelectric points (*pI*) varied from 5.34 (*LcCCR4*) to 7.52 (*LcCCR3/5*). The average amino acid number, MW, and *pI* of these protein sequences were 330, 36309.11 Da, and 6.33, respectively. We used WoLF POST and online analysis with the TargetP-2.0 Server to predict the subcellular localization of the LcCCR proteins. Both online tools indicated that LcCCR proteins are localized in the cytoplasm or the chloroplast (**Table 1**).

**Table 1:** The characteristic of the 13 *LcCCR*s in *L. chinense*.

	Amino acid						Subcellular
Gene Name	Gene ID	Length	CDS	Exon	*MW*(Da)	*pI*	Localization
*LcCCR1*	Lchi22380	337	1014	5	37172.84	7.19	chloroplast
*LcCCR2*	Lchi22382	337	1014	5	36971.62	7.19	chloroplast
*LcCCR3*	Lchi22384	336	1011	5	37022.64	7.52	chloroplast
*LcCCR4*	Lchi06756	261	786	7	28933.98	5.34	chloroplast
*LcCCR5*	Lchi06755	336	1011	6	37022.64	7.52	cytoplasmic
*LcCCR6*	Lchi19772	274	825	5	30552.23	5.74	cytoplasmic
*LcCCR7*	Lchi17241	329	990	6	36205.31	5.78	chloroplast
*LcCCR8*	Lchi32967	368	1107	6	40758.87	7.24	chloroplast
*LcCCR9*	Lchi27808	324	975	6	35443.78	6.20	cytoplasmic
*LcCCR10*	Lchi27811	324	975	6	35454.83	5.86	cytoplasmic
*LcCCR11*	Lchi00871	395	1188	6	43564.06	5.48	cytoplasmic
*LcCCR12*	Lchi25817	326	981	6	35868.37	5.46	chloroplast
*LcCCR13*	Lchi25815	338	1017	6	37047.31	5.77	chloroplast

The number of amino acids represents the number of amino acids in the protein encoded by the LcCCRs. CDS is the sequence that encodes the protein products. MW represents the molecular weight of the protein encoded by the LcCCRs. pI represents the isoelectric point of the protein encoded by the LcCCRs.

The subcellular localization was predicted by online tools: WoLF PSORT (https://wolfpsort.hgc.jp/) and TargetP-2.0 Server (http://www.cbs.dtu.dk/services/TargetP/). The physical and chemical characteristics of LcCCR proteins identified in the *L. chinense* genome were predicted using the online tool, ProtParam (https://web.expasy.org/protparam/).

### Multiple sequence alignment and phylogenetic analysis of the LcCCR gene family

**Figure 1:**
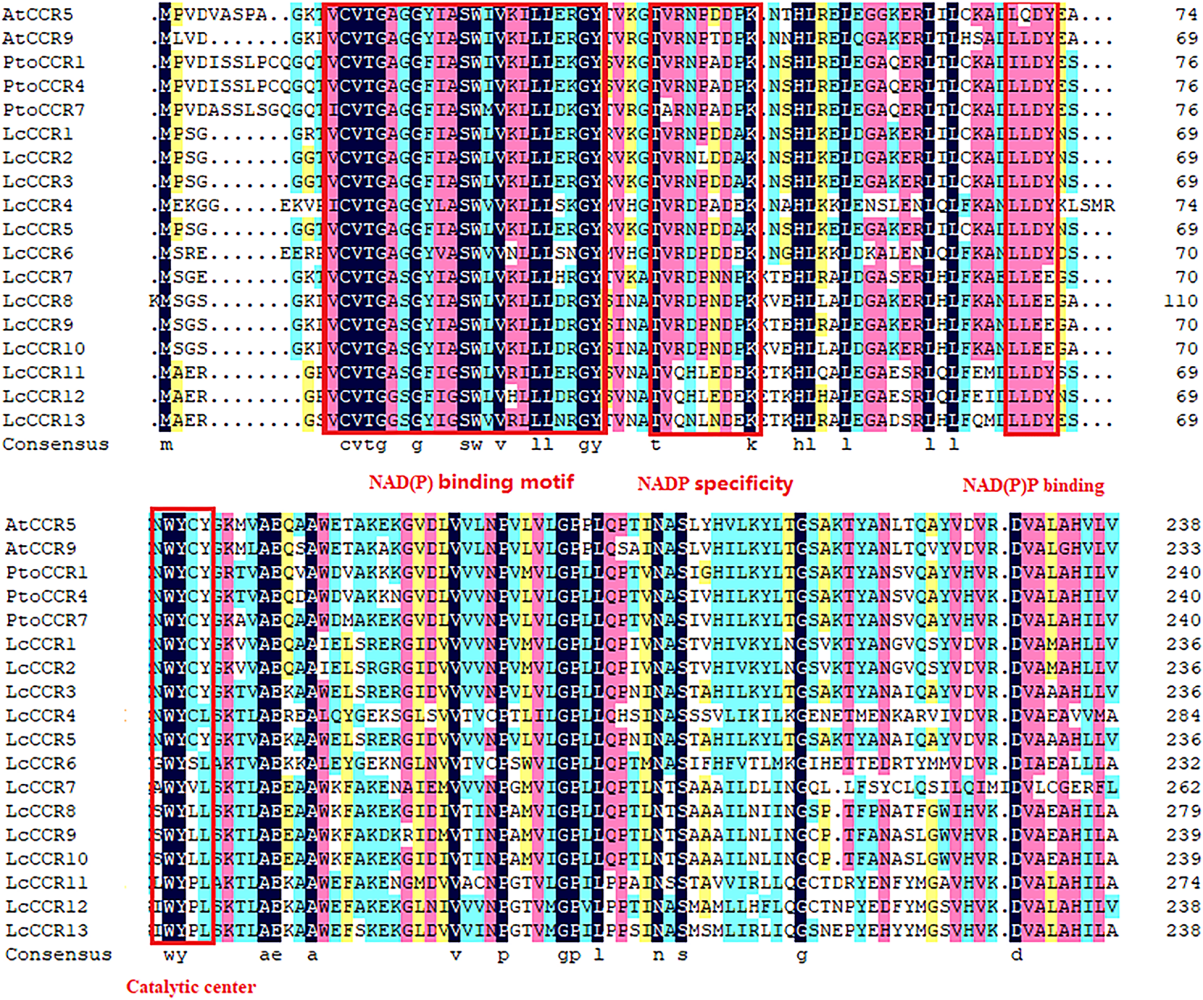
Characteristics of the amino acid sequences of LcCCRs (LcCCR1-13) and other CCR proteins (AtCCR5: AT1G15950; AtCCR9: AT1G80820; PtoCCR1: KP281597; PtoCCR4: KP281599; PtoCCR7: KF145198). Multiple sequence alignment was performed by DNAMAN (version 6.0). The conservative functional domains: NAD(P) binding motif (VCVTGAGGXIASWXVKXLLXXGY), NAD(P) specific site (TVRNPDDPK), NAD(P) binding (LLLDY), and catalytic center were surrounded by red boxes (NWYCY).

To understand the sequence characteristics, a multiple sequence alignment analysis of the 13 LcCCR proteins was performed by DNAMAN software with default parameters. Two AtCCR proteins (AtCCR5/9) and three PtoCCR proteins (PtoCCR1/4/7) were selected as representatives for further comparison. The conserved domain structures in LcCCRs are displayed in **Figure 1**. It was predicted that the amino acid sequences in each member of the LcCCRs were significantly different from other CCR proteins, but the C-terminal and the middle sequences were highly conserved. Almost all of the above sequences contained the conserved domain, “X-W-Y-X-X,” from the cinnamyl-CoA reductase family of proteins and the recognition domain of NAD(P)-dependent short-chain reductase (SDR). The structure also included the typical “Rossmann fold” and the conserved catalytic center, the Ser-Tyr-Lyr triad (Kallberg et al., 2002).

**Figure 2:**
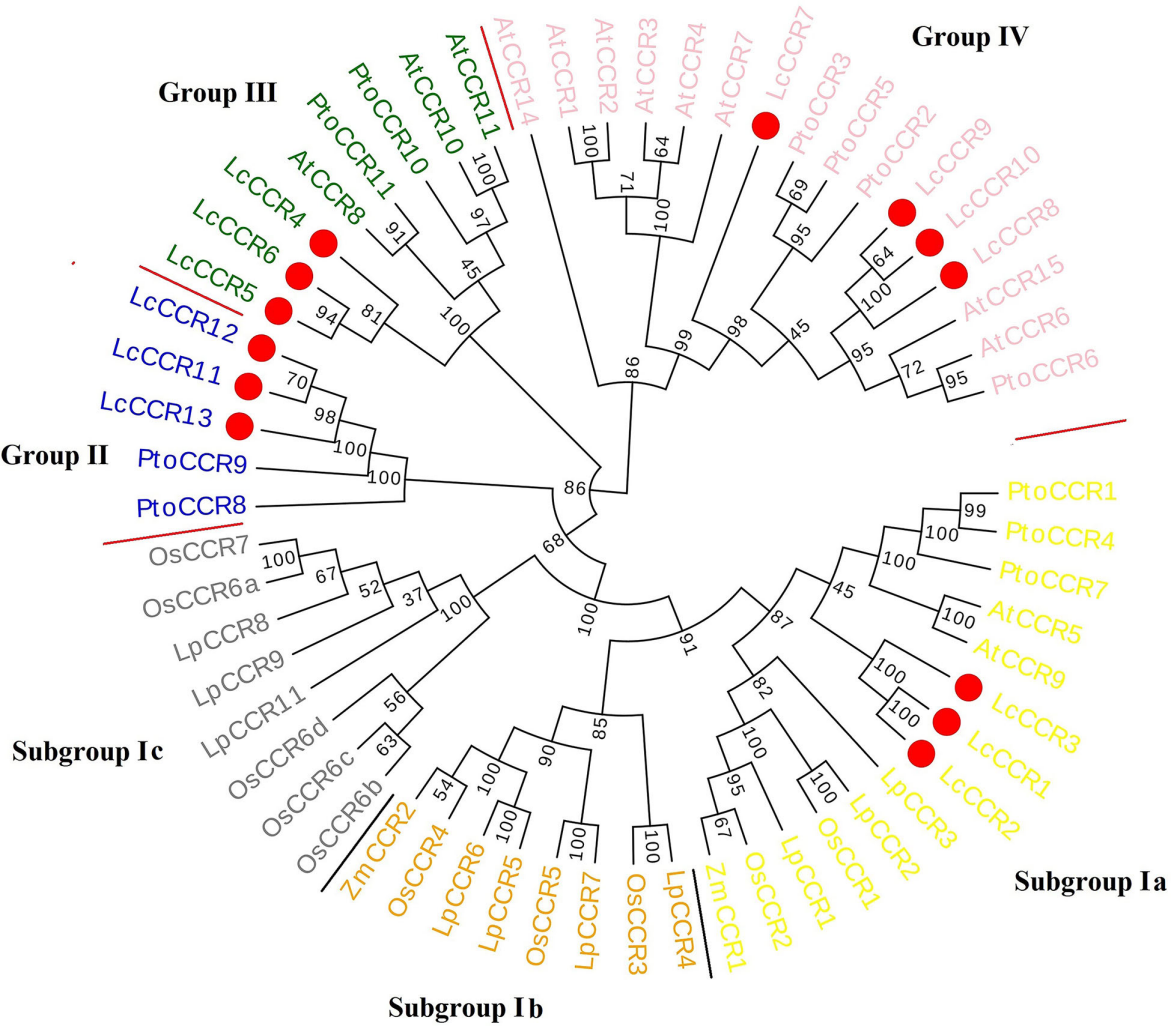
Phylogenetic tree of CCR protein sequences from various plant species. Circular phylogenetic tree of CCR protein sequences of various plants. The phylogenetic tree consisted of 59 CCR proteins (13 LcCCRs, 13 AtCCRs – *A. thaliana*, 11 PtoCCRs – *P. tomentosa*,10 LpCCRs – *L. perenne*, 10 OsCCRs – *O. sativa*, 2 ZmCCRs – *Z. mays*). Protein sequences were aligned and a phylogenetic tree was constructed using MEGA5.1 with the maximum likelihood method and visualized by Evoview software. Substitution and site change rates were calculated using the Jones–Taylor–Thornton (JTT) model and the Gamma distributed with Invariant sites (G+I) model. Bootstrap analysis was performed on 1000 replicates to calculate the reliability of the phylogenetic tree. Bootstrap values are shown at each branch as percentages. The red circle indicated LcCCR protein sequences; different color backgrounds and red lines distinguish the different groups. The phylogenetic tree consists of four groups: Group I (Subgroup Ia – yellow, Subgroup Ib – orange, Subgroup Ic – grey), Group II – blue, Group III – green, and Group IV – pink. The higher value on the branch of the evolutionary tree, the higher reliability of this branch.

To clarify the phylogenetic relationship between the LcCCRs and other CCR proteins, an unrooted phylogenetic tree was constructed with 59 CCR proteins (13 LcCCR, 13 AtCCR, 11 PtoCCR, 10 LpCCR, 10 OsCCR, and 2 ZmCCR proteins) based on the maximum likelihood method. In the phylogenetic tree, all 59 CCRs mentioned above could be roughly divided into four groups (Groups I to IV; **Figure 2**). Our results showed that Group I was divided into three subgroups: subgroups Ia, Ib, and Ic. Subgroup Ia contained 14 CCR members (3 LcCCRs, 2 AtCCRs, 3 LpCCRs, 3 PtCCRs, 2 OsCCRs, and 1 ZmCCRs), which were related to lignin biosynthesis [AtCCR5/9 (Goujon et al., 2003), ZmCCR1 (Pichon et al., 1998), PtCCR1/4/7 (Chao et al., 2017)]. The entire Group I comprised CCRs from dicotyledons and monocotyledons. Subgroup Ib contained 8 CCR members (4 LpCCRs, 3 OsCCRs, and 1 ZmCCR). Subgroup Ic contained 8 CCR members (3 LpCCRs and 5 OsCCRs). Subgroups Ib and Ic consisted only of CCR proteins from the monocotyledons. Group II, shown in blue color, contained 5 CCR members (3 LcCCRs and 2 PtoCCRs). Group III in green contained 8 CCR members (3 LcCCRs, 3 PtoCCRs, and 2 AtCCRs). Group IV in pink contained 16 CCR members (4 LcCCRs, 4 PtoCCRs, and 8 AtCCRs) (**Figure 2**). Notably, most CCRs were evenly distributed in four groups (Barakat et al., 2011). Moreover, it was found that the *LcCCR*s had close phylogenetic relations with their ancestors’ species in each group. The distribution of CCR proteins in the phylogenetic tree suggested that CCR proteins were duplicated multiple times before becoming specific to the monocotyledon and dicotyledon species in which they were observed.

### Gene structures and conserved motif composition in the LcCCR gene family

**Figure 3:**
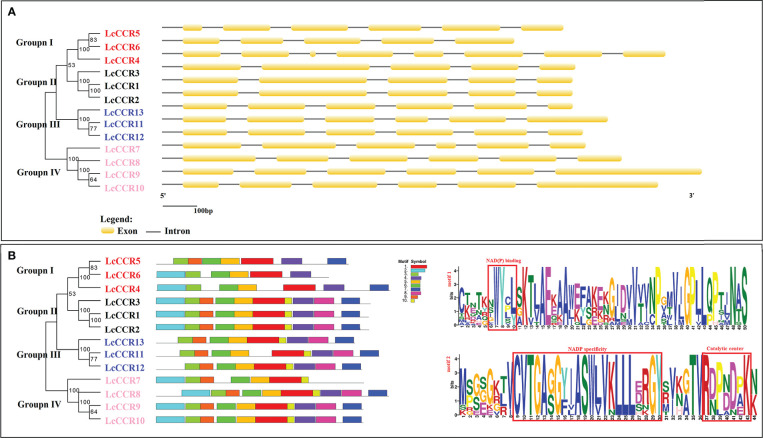
Map of the gene structures and conserved motifs patterns in *L. chinense* CCR proteins. The phylogenetic relationship was based on *LcCCRs*. According to phylogenetic relationships, 13 *LcCCR*s were divided into four distinct groups (I–IV). Different subfamilies were represented by different colors and names. The red background denotes Group I; The grey color background denotes group II; The blue background denotes Group III; The orange color background denotes Group IV. **(A)** The exon–intron structure (visualized by the GSDS online tool) of LcCCR proteins. The yellow squares indicate exons, and the black lines indicate introns. **(B)** Distribution of 10 conserved motifs (visualized by MEME online tool) in the LcCCR proteins. Differently colored squares indicate different motifs. The bottom scale shows the protein length, and the details of two crucial conserved motifs in the LcCCR proteins are shown at the back of the scale. Motif 1: CKETKNWYCLSKTLAEKAAWEFAKEKGJDVVTVNPGMVJGPLLQPTJNAS, contains the NAD(P) binding; Motif 2: MPGEGKTVCVTGAGGYIASWLVKLLLERGYSVKGTVRDPBDPKN, contains the NAD(P) specific site and the catalytic center. More detail of the motif sequences is presented in **Table S3**.

The exon-intron configurations in the *LcCCR* genes were examined to further explore the probable structural evolution of this family of genes (**Figure 3A**). Similar structures were usually observed in the same group. e.g., the members of Group II contained 5 exons, and the members of Group III/IV contained 6 exons. The length and number of introns were generally similar in each group, which was consistent with the clusters of *LcCCR*s. However, there were some exceptions. For example, the members of Group I did not have a similar exon-intron pattern.

Furthermore, we investigated the full-length protein sequences of the 13 *LcCCRs* to identify their conserved motifs. 10 conserved motifs were predicted to be present in 13 LcCCRs. The number of amino acids in the 10 motifs ranged from 11 to 50 (**Table S3**). The patterns of the conserved motifs are shown in **Figure 3B**. As motif 1 constituted the main structure of the CCR domain (“NWYCY”), it was identified in all LcCCR proteins. LcCCR proteins in the same group tended to have similar motif compositions, suggesting that protein structures were conserved in a specific subfamily (Song and Peng, 2019). Furthermore, only the members of Group I of LcCCR proteins and LcCCR7 did not contain Motif 8. We speculate that LcCCR7 may have a deletion, leading to the lack of this discrepancy. Overall, members within each group shared the same genetic structure, which was consistent with their phylogenetic relationships. The stability of group classifications was maintained as per our findings from surveys on the conserved motif compositions, gene structures, and phylogenetic relationships.

### *Cis*-acting element prediction in the promoters of 13 *LcCCR* genes

**Figure 4:**
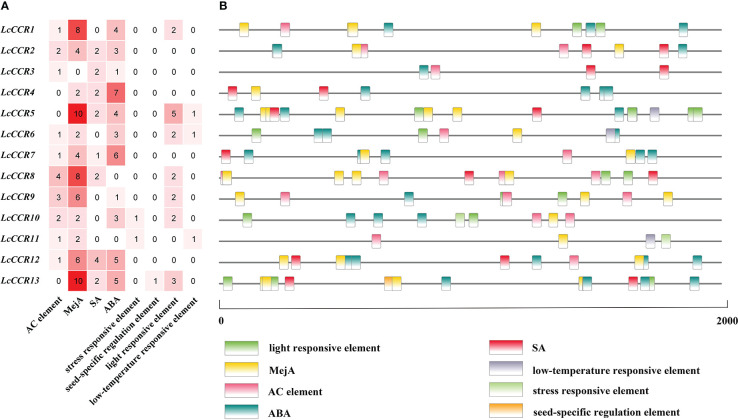
Analysis of cis-elements in *LcCCR* promoters (2000 bp upstream of the translation start site). **(A)** Number of distinct cis-elements in each *LcCCR* promoters. **(B)** location of distinct cis-elements in each *LcCCR* promoters. The figure shows the *cis*-elements that were related to different stress and hormone responses in the putative promoters of *LcCCR*s. All *LcCCR* gene promoters mainly contained the following eight elements: AC element, MejA (cis-acting regulatory element involved in the MeJA-responsiveness), SA (cis-acting element involved in salicylic acid responsiveness), ABA (cis-acting element involved in the abscisic acid responsiveness), stress responsive element, seed-specific regulation element, light-responsive element, and low-temperature element. Detailed information on c*is*-acting elements is given in **Table S4**.

To explore gene function and regulation patterns, we surveyed the *cis*-elements in a region 2000 bp upstream of the initiation codon in each *LcCCR* gene. The *cis*-acting elements of the *LcCCR* genes were then predicted by searching the promoter sequences from the PlantCARE database (Song and Peng, 2019). As shown in **Figure 4**, almost all *LcCCR* gene promoters contained an AC element (Raes et al., 2003), along with the methyl jasmonate (MejA), salicylic acid (SA), and abscisic acid (ABA) elements. Some promoters contained light response seed-specific regulation (Chen et al., 2020b) or low-temperature response elements. The number of *cis*-acting elements for each of the 13 *LcCCR* genes ranged from 4 to 22. Promoters of different genes in the same group contained similar elements with only minor differences.

The promoter sequences of most genes (70%) had AC elements, such as *LcCCR1/2/3/6/7/8/8/10/11/12* (**Figure 4A**), suggesting that these genes may be involved in the regulation of phenylpropanol metabolism and other unknown functions. In addition, these *cis*-elements are evenly distributed in the promoter region of *LcCCRs* (**Figure 4B**).

*Cis*-acting elements, such as ABRE, TCA-element, and TGACG-motif, are related to the signaling pathways of ABA, SA, and MejA, respectively. All of these metabolites are related to stress resistance in plants. Almost all promoters contain these three *cis*-acting elements, therefore, it can be reasonably inferred that some *LcCCR*s might be involved in plants’ responses to biotic and abiotic stresses.

Other promoters contained *cis*-elements for seed-specific regulation, light response, and low-temperature response, indicating that they may have a certain influence on the seed. The low-temperature response was related to the light response. Detailed information is shown in **Table S4**.

### Tissue expression patterns of *LcCCR*s revealed by RNA-seq data

**Figure 5:**
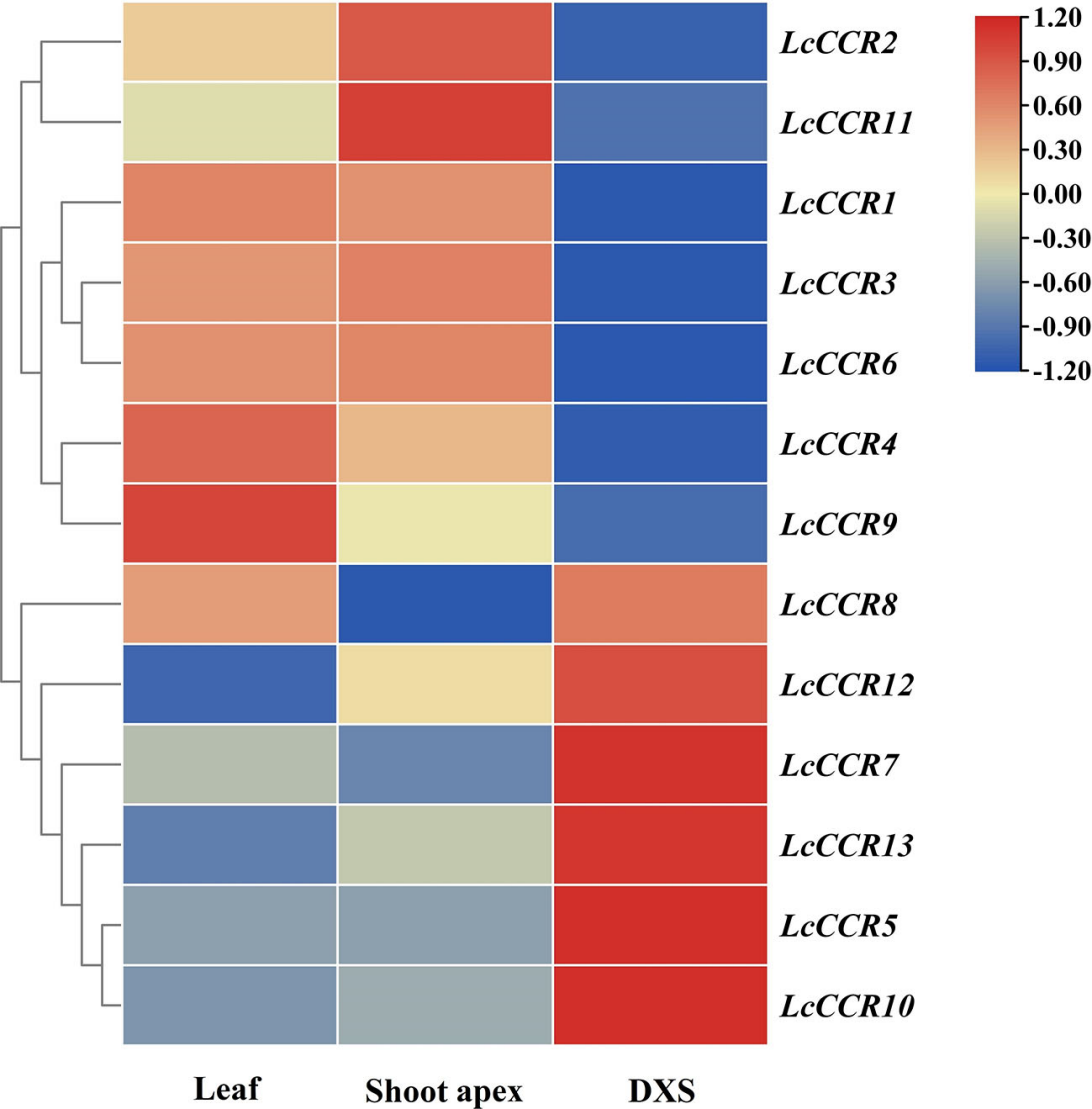
Tissue expression patterns of 13 *CCR* genes across various tissues in *L. chinense* based on FPKM values from transcriptomic data. Heatmap showing the relative expression levels of each *LcCCRs* in three tissues, including leaf, shoot apex, and DXS (developing xylem at stem) (based on RNA-seq data which will be released in another paper). FPKM (fragments per kilobase of transcript per million fragments mapped) values shown were standardized by the Z-score, and the heatmap was constructed by TBtools (version 1.0) software. The x-axis shows the different tissues, and the y-axis shows the different genes. The red box indicates a relatively high expression level, while the blue box indicates a relatively low expression level.

We investigated the expression patterns of *LcCCR*s in different tissues (leaf, shoot apex, and DXS) with Illumina RNA-seq data (Unpublished data from our laboratory). The results showed that the 13 *LcCCR* genes were expressed in all three tissues but some genes exhibited tissue-specific expression. For instance, *LcCCR1/3/4/6/9* were highly expressed in the leaf. While in the shoot apex, the expression levels of *LcCCR2/11* were the highest. Meanwhile, *LcCCR8* displayed high expression in the leaf and DXS. Furthermore, it was found that *LcCCR5/7/10/12/13* had the highest expression in the DXS (**Figure 5**). Collectively, the different expression patterns of *LcCCR*s suggest that they may play different roles in the various developmental stages/tissues in *L. chinense.*

### Tissue expression specificity analysis of *LcCCR5/7/10/12/13*

**Figure 6:**
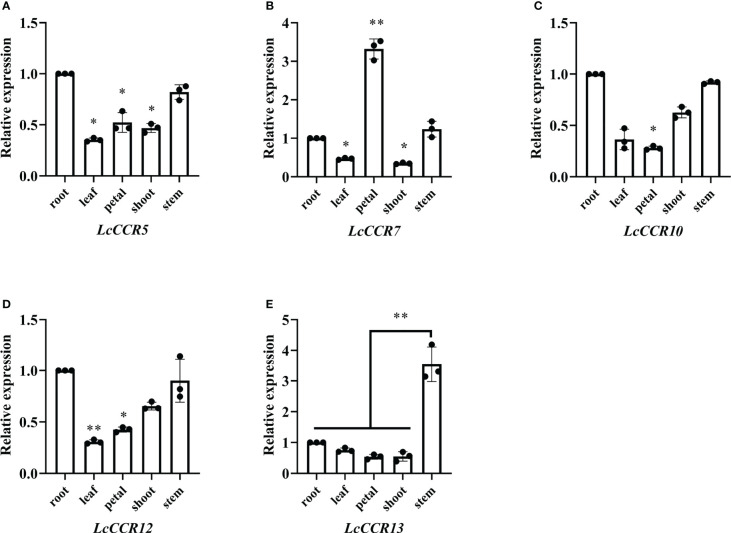
Tissue expression specificity analysis of *LcCCR5/7/10/12/13*. **(A–E)** The x-axis represents different tissues (root, stem, leaf, petal, and shoot). The value of gene expression in root was set to 1. *Tubulin* was used as the internal control. Tissue-specific expression analyses were performed using real-time qPCR; the values are presented as means ± SD from three biologically independent replicates (each biological replicate had three technical replicates). Gene expression profiles were evaluated by the 2^−ΔΔCT^ method. The y-axis represents the relative expression level. Asterisks above the bars in figures indicate significant differences (Student’s t-test; *p < 0.05, **p < 0.01) as compared to the roots (*LcCCR5/7/10/12*)/stems (*LcCCR13*). The raw data are marked as a point on the histogram. The error bars in the charts indicate the standard deviation from the mean of the triplicate treatments.

To obtain insights into the potential roles of *LcCCR5/7/10/12/13*, which were predicted to be highly expressed in DXS (**Figure 5**), the expression level of *LcCCR* genes was further determined by RT-qPCR in five tissues (root, stem, leaf, shoot, and petal). The results showed that the *LcCCR5/7/10/12/13* were expressed in all the tested tissues, but their expression levels were different (**Figure 6**). The relative expressions of *LcCCR5/10/12* were similar in all tissues with the highest expression level in the root. Moreover, the relative expression level of *LcCCR7* in the petal was the highest. Furthermore, *LcCCR13* had the highest expression level in the stem, too, which was extremely significantly different from the expression in the root, leaf, petal, and shoot. Although the expression of the *LcCCR13* gene was not xylem-specific, it was mainly expressed in lignin-forming tissues, such as stem, suggesting that *LcCCR13* may play an important role in lignin biosynthesis in *L. chinense.*

### Growth and morphological characteristics of *LcCCR13* transgenic tobacco

**Figure 7:**
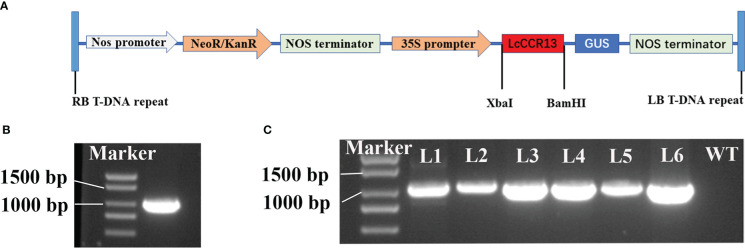
The genetic transformation of the *LcCCR13* gene. **(A)** Schematic diagram of the overexpression vector construction. *35S*: cauliflower mosaic virus *35S* promoter. GUS: *β*-glucuronidase. Nos terminator: nopaline synthase gene terminator. KanR: Kana resistance gene. RB and LB: the right and left borders, respectively. **(B)** ORF cloning of the *LcCCR13* gene, and the length of *LcCCR13* was 1017 bp. **(C)** Verification of *LcCCR13* tobacco transgenic lines by PCR amplification. DNA was extracted from the leaves of 2-month-old tobacco plants. The marker length was 2000 bp.

**Figure 8:**
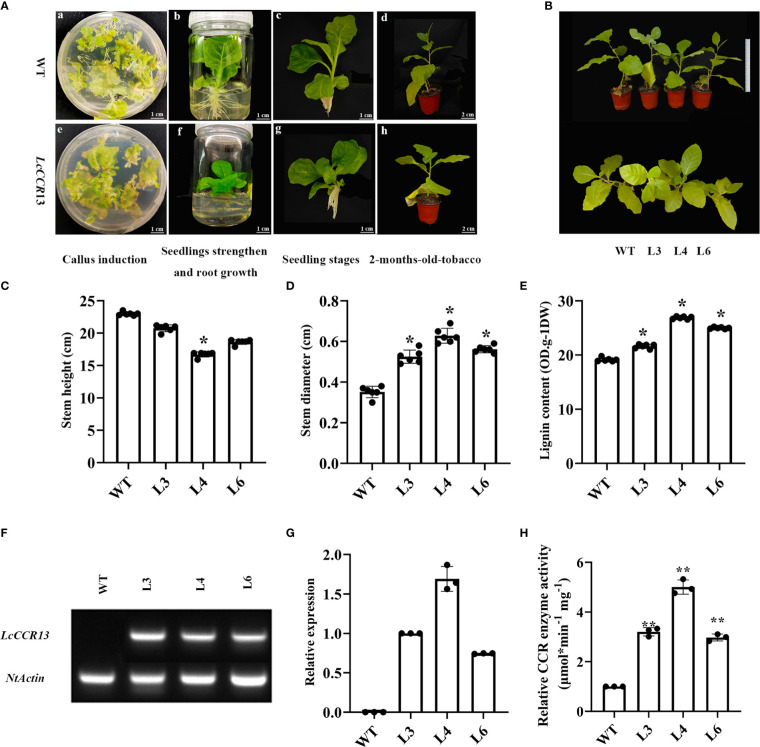
The phenotypic and physiological traits of transgenic tobacco. **(A)** The different growth stages of the WT and transgenic tobaccos. The a–d figures show the representative images of wild-type (WT), and the e–f figures show the representative images of *LcCCR13* transgenic tobaccos. The a and e figures show the periods of callus induction. The b and f figures show the periods of seedlings’ strengthening and root growth. The c and g figures show the seedling stage. The d and h figures show the periods of 2-month-old tobacco. Four diverse mediums included: differentiation medium (MS + 0.05 mg/L NAA + 2.0 mg/L 6-BA + 40 mg/L Kan + 150 mg/L TMT), selective medium (MS + 0.05 mg/L NAA + 2.0 mg/L 6-BA + 40 mg/L Kan + 200 mg/L TMT), seedling strengthened medium (MS + 0.05 mg/L NAA + 2.0 mg/L 6-BA + 40 mg/L Kan + 250 mg/L TMT), and root growth medium (1/2 MS + 250 mg/L TMT), respectively. Bar = 1 cm (a-c, e-g); Bar = 2 cm (d and h). **(B)** The growth conditions of the WT and the transgenic tobacco lines (L3, L4, L6) for 3 months. Bar = 20 cm. **(C)** Stem height, **(D)** Stem diameter, and **(E)** lignin content of the WT and 2-month-old transgenic tobacco lines from six biological repeats. **(F)** A semi-quantitative (*NtActin* was detected in all the transgenic lines and WT; *LcCCR13* was detected in all the transgenic lines except WT) **(G)** RT-qPCR assay of *LcCCR13* with WT, L3, L4, and L6 lines (the expression level of *LcCCR13* in L3 was set to 1). **(H)** The relative activity of CCR enzyme in WT and transgenic plants (The activity of CCR of WT was set to 1, and the value of L3/4/6 CCR activity were calculated when compared to the value of WT). There were three biologically independent replicates, where each biological replicate had three technical replicates. Error bars in the charts indicate the standard deviation from the mean of each repeat. Asterisks indicate significant differences between the transgenic tobacco and WT plants (Student’s t-test; *p < 0.05, **p < 0.01). The data were the Mean ± standard error.

To further investigate the function of the *LcCCR13* gene in lignification, a transgenic assay was used. Tobacco leaves were transformed with the *35s::LcCCR13* vector to induce high expression of the gene (**Figures 7A, B**); six *LcCCR13* positive lines were thus obtained (**Figure 7C**). To investigate the influence of the *LcCCR13* gene on plant growth, the WT controls and the three transgenic tobacco lines were monitored. Significant differences were observed in the growth phenotypes of the transgenic lines and the WT plants (**Figures 8A, B**). For example, there was a significant height difference between the L4 lines and the WT plants (0.7 fold; **Figure 8C**). We found that the transgenic lines had a significantly greater stem diameter than the WT (1.6 fold; **Figure 8D**). All in all, transgenic lines exhibited altered growth characteristics as compared to the WT plants.

### *LcCCR13* overexpression positively regulates lignin synthesis

A significant increase was noted in the lignin content of the transgenic lines (1.3 fold; **Figure 8E**), and the L4 lines were selected for histochemical staining due to the highest *LcCCR13* gene relative expression in them (**Figures 8F, G**). The difference in the CCR activity of transgenic lines was measured. We found that the relative activity of CCR in transgenic plants was significantly higher than that in the WT plants, and the L4 lines had the highest CCR activity compared to other transgenic lines (**Figure 8H**).

**Figure 9:**
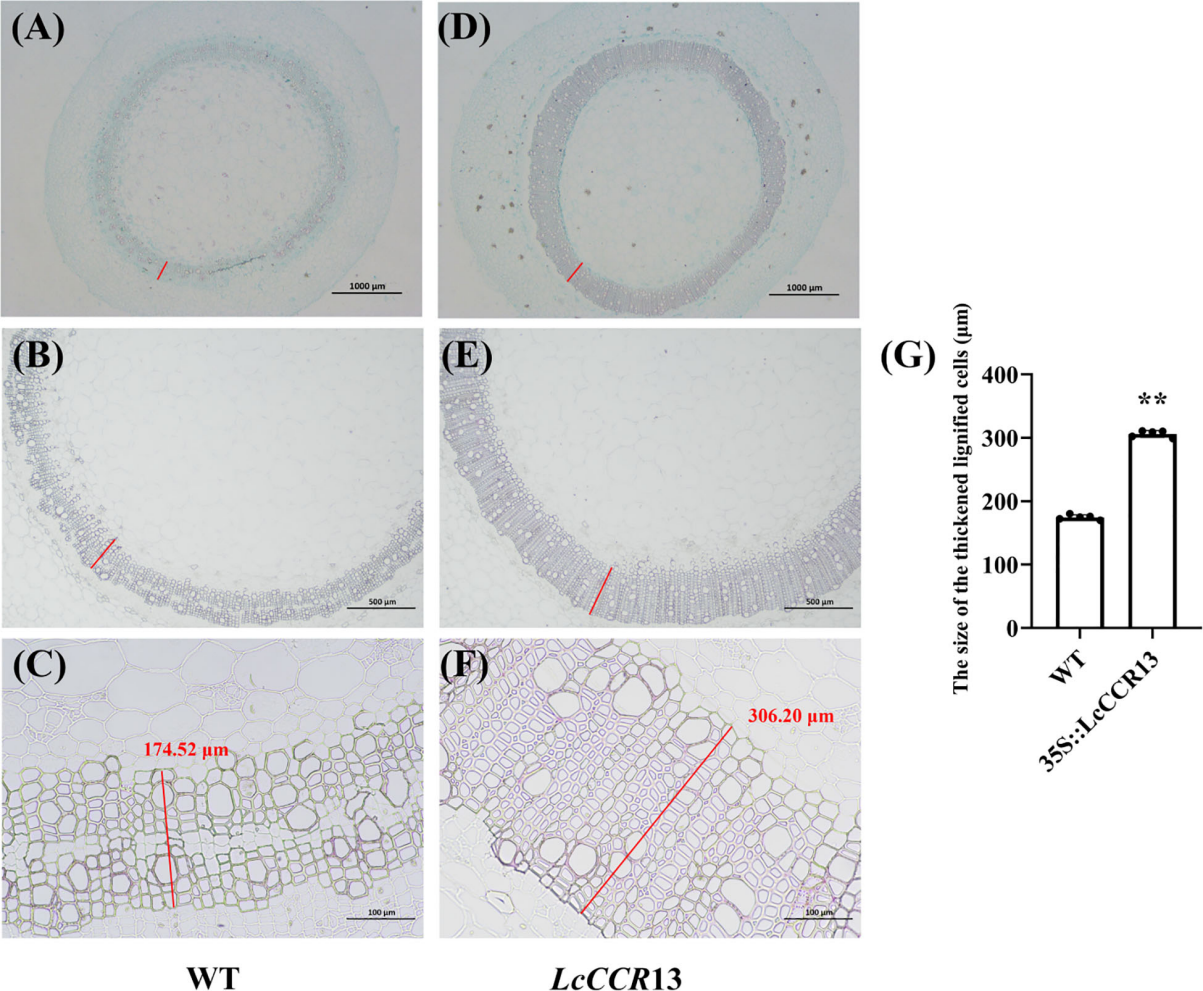
Sectioned and microscopic observation of the wildtype (WT) and transgenic tobacco stem. Sections stained with phloroglucinol-HCl and safranin of transgenic tobacco stems. **(A-C)** WT tobacco; **(D-F)** Transgenic tobacco lines (L4). The brown region represents the site of lignin deposition. **(A, D)** Cross sections of 2-month-old L4 and WT plants with the 2^nd^ internode (IN). The cross sections were stained with safranin O and Fast Green. Bar, 1000 μm. **(B, C, E, F)** Cross sections of 2-month-old L4 and WT plants with the 2^nd^ internode (IN). The cross sections were stained with phloroglucinol-HCl. Bar, 1000 μm **(B, E)** and 100 μm **(C, F)**. The red lines were the size of the thickened and lignified cells, and the number in the figure denotes the length (μm, measured by ImageJ, version 2.3.0). **(G)** The size of the thickened and lignified cells (μm) in WT and transgenic tobacco. Error bars in the charts indicate the standard deviation from the mean for the three repeats. Asterisks indicate significant differences between the transgenic tobacco and WT plants (Student’s *t*-test; *p < 0.05, **p < 0.01). The data are the mean ± standard error.

When observed under higher magnification, the tissue sections of the L4 lines showed a more highly developed xylem with wider and deeper brown color than in the WT plants (**Figures 9A–F**). Furthermore, the size of the thickened and lignified cells in transgenic tobacco was 306.20 μm, and they were ~1.75× (*p* < 0.01) as thick as the WT cells (174.52 μm; **Figure 9G**). Therefore, higher lignin deposition occurred in the L4 lines than in the WT plants. These results suggest that the overexpression of *LcCCR13* might positively regulate lignin synthesis.

## Discussion

### Sequence characteristics and differential analysis of *LcCCR*s in *L. chinense* revealed functional differentiation

Lignin synthesis starts from the common phenylpropanoid pathway and is regulated *via* a variety of enzymes, but the cinnamyl-CoA reductase encoded by the *CCR* is one of the key enzymes in lignin biosynthesis (Lewis and Yamamoto, 1990; Raes et al., 2003). As for most forest tree species and associated metabolic pathways, genome-wide analysis is a crucial method to elucidate the biological functions of the *CCR* family in plants. Here, we report the phylogeny and genome structure of the *CCR* genes in basal angiosperms for the first time. After further characterization, 13 *LcCCR* genes were identified in *L. chinense*.

The expansion of the CCR family members is important to plant evolution. After years of continuous research, studies have identified and cloned the full-length or partial coding sequence (CDS) of *CCR* genes from the xylem or other tissues in many plant species. For example, seven *CCR* genes involved in lignin biosynthesis have been identified in *P. tomentosa* (Chao et al., 2017), 11 in *Populus tremuloides* (Li et al., 2006), 4 in *Boehmeria nivea* (Tang et al., 2019), and 11 in *A. thaliana* (Raes et al., 2003). In general, there are more *CCR* genes in woody species. We hypothesize that the number of CCR family members in different species may be affected by genomic differences, the redundancy of gene functions, or species differences.

In this study, 13 *LcCCR* genes were screened by proteomic mining of the complete sequence genome of *L. chinense*. The molecular weight of these LcCCR proteins was greater than 35 kDa each, which is the same as most CCR proteins reported in plants (Prasad et al., 2010). Their isoelectric points were between 5.5 and 7.5, which is consistent with those for the BnCCR proteins (Tang et al., 2019). Lauvergeat et al. examined the protein structures of the bona fide Arabidopsis CCR proteins, AtCCR5 (AT1G15950) and AtCC*R*9 (AT1G80820) (Lauvergeat et al., 2001), to reveal that the region of the N-terminal portion of AtCCR5/9 involved in the NADP(H) cofactor binding site was conserved. A very well-conserved motif, KNWYCY, which is thought to be involved in the catalytic site of this enzyme, also exhibits the signature of CCRs (Lacombe et al., 1997). Previous studies have shown that the second and third amino acids (“W” and “Y”) are crucial for the binding of the enzyme to the substrate, and thus, they are rarely replaced (Barakat et al., 2011). The conserved domain, “X-W-Y-X-X”, in the cinnamyl-CoA reductase family of proteins was found in all 13 LcCCR protein sequences here. A similar phenomenon was observed in *Pyrus bretschneideri* (Chen et al., 2020b) indicating that *LcCCR*s remained highly conserved during the evolution of *L. chinense.*

Based on the results of MEME analysis with default parameters, the same group of LcCCR proteins shared common motifs in the phylogenetic tree, suggesting that these LcCCRs are highly conserved, strongly supporting the reliability of the group classifications (Bailey et al., 2006). In addition, gene structure and motif analysis methods were employed to discover the potential features of genes (Barakat et al., 2011). Phylogenetic analysis shows that *LcCCR* genes could be divided into four groups. The structures of exon-intron regions in the genes from the same group were similar. The change in the number of *CCR* genes and the diversification of characteristic motifs provides new insights to understand the evolution and gene function of the LcCCR gene family.

### Role of the *LcCCR13* gene in the lignin biosynthesis

The phenylpropanoid pathway, the main pathway in lignin biosynthesis that is regulated by a variety of enzymes, is well understood. Cinnamoyl-CoA reductase (CCR) is considered to be the first committed enzyme in the lignin-specific branch because it can catalyze the conversion of cinnamoyl-CoA to cinnamaldehyde in the monolignol biosynthetic pathway, which can further be transferred into three lignin monomers (G, S, and H) (Gayoso et al., 2010). Besides, cinnamoyl-CoA can be synthesized into phenolic substances (such as anthocyanins and flavonoids) when the function of CCR has been lost. For this reason, the key role of the *CCR* gene in lignin synthesis has been confirmed in many plant species, and changing its expression levels might significantly affect the lignin content and growth of plants (Liu et al., 2021). In our study, we measured the plant height and the lignin content in transgenic lines and observed that the overexpression of the *LcCCR13* gene significantly increased lignin accumulation in the stems and decreased plant height. These results corroborate that the *LcCCR13* is a key gene for lignin synthesis in the stems of *L. chinense*.

The synthesis of lignin is tissue specific. In general, lignin synthesis occurs in tissues with higher lignification, and the *CCR* genes are strongly expressed in such tissues. For example, *AtCCR5* is mainly concentrated in the tissues that are being lignified and participate in the process of tissue lignification (Lauvergeat et al., 2001; Goujon et al., 2003); the activity of *PtoCCR1/4/7* affects the accumulation of lignin (Chao et al., 2017). The relative expression levels of the *EgCCR* gene are also the highest in the stems, followed by their expression in the roots and other tissues (Lacombe et al., 1997). *TaCCR2* is mainly expressed in the roots but also participates in the lignin synthesis in stems, indicating that it plays an important role in lignin synthesis in *T. aestivum* (Lin et al., 2001). The expression analysis of 10 *PoptrCCR*s showed that all of them were expressed in the bark, leaves, and xylem, but only the bona fide *PoptrCCR12/14* had the highest expression levels in the xylem, where they showed a significant difference from the expression levels in the leaf/bark (Barakat et al., 2011). Our transcriptome data indicated that only *LcCCR5/7/10/12/13* genes were predominantly expressed in the DXS (stem developmental xylem). RT-qPCR assays showed that the *LcCCR13* had the highest expression level in the stem, which was significantly different from the expression level in other tissues. These observations were largely consistent with previous studies; For instance, the bona fide *CCR* genes are highly expressed in the tissues/organs with high lignification (Zhang et al., 2015). In this study, only *LcCCR13* was highly expressed in the stem, indicating that it might play potential roles in stem development. Therefore, we overexpressed *LcCCR13* in tobacco to determine whether it was associated with lignin synthesis.

Transgenic technologies have been widely used to control the lignin content and composition in various plants. Up-regulation or down-regulation of *AtCCR* expression in *A. thaliana* significantly affected the lignin content and other characteristics related to plant growth and development (Goujon et al., 2003). As compared to the WT plants, the transgenic lines of *B. napus* over-expressing *BnC.CCR2b* had significantly higher lignin content in the stems (Prasad et al., 2010). When BnCCR1 activity was increased in *B. platyphylla*, the lignin content also increased, and the height of the transgenic plants was reduced (Zhang et al., 2015). The lignin content increased and the plant height decreased in the antisense transgenic tobacco plants, and their plant phenotypes (such as plant height, seed quality, and length of the leaves) was significantly altered (Chabannes et al., 2001). These findings indicate that the manipulation of *CCR* gene expression affects lignin content, and the changes in lignin content in the transgenic plants is incompatible with normal phenotypes.

In this study, we found that the over-expression of the *LcCCR13* gene affects the growth and development of transgenic tobacco. As compared to other transgenic lines, transgenic plants with the highest *CCR* activity showed the highest lignin content, indicating that *CCR* might be the key gene involved in lignin biosynthesis. Hamedan et al. also reported that increased lignin content coincides with CCR activities in *Gerbera jamesonii* (Hamedan et al., 2019). Moreover, according to the measurement of lignin content and its deposition sites in transgenic plants, we conclude that the *LcCCR13* gene has a significant effect on lignin synthesis in the stem. Based on the fact that increasing the expression of *LcCCR13* reduced the height growth and increased the lignin content in the stem, we assume that *LcCCR13* is involved in the thickening of the cell wall by increasing the levels of all three types of subunits. It further regulates the ligin content in plants. Tu et al. found that the downregulation of *CCR1* expression in transgenic *L. perenne* plants reduced the lignin content by lowering the levels of all three lignin monomers (Tu et al., 2010). Interestingly, Zhang et al. found that *BpCCR1* could control the height growth and lignin content by lignifying the cell wall (Zhang et al., 2015), which was consistent with our results. All in all, these findings might be significant for understanding the roles of the *LcCCR13* gene in lignin biosynthesis in the stems of *L. chinense*.

## Conclusions

In this study, 13 *LcCCR* genes were identified in the *L. chinense* genome, among which the *LcCCR13* is speculated to potentially play a role in lignin synthesis in the stem as per the results of phylogenetic and bioinformatics analysis, gene expression profiling *via* RT-qPCR assays, and function verification *via* gene transformation in tobacco. In conclusion, this study lays a foundation to uncover the mechanism of wood formation in *L. chinense*.

## Data availability statement

The datasets presented in this study can be found in online repositories. The names of the repository/repositories and accession number(s) can be found in the article/**Supplementary Material**.

## Author contributions

WL designed the experiments, performed the experiments, analyzed the experimental data, and wrote the paper. ZH, LY and JW collected plant materials and formally analyzed the experimental data. HX and ZT performed the experiments and formally analyzed the experimental data. ZC collected plant materials and analyzed the experimental data. HL conceived and designed the experiments, gave comments on the data analysis, and revised the paper. All authors contributed to the article and approved the submitted version.
